# Brescia-COVID Respiratory Severity Scale (BRCSS) and Quick SOFA (qSOFA) score are most useful in showing severity in COVID-19 patients

**DOI:** 10.1038/s41598-021-01181-x

**Published:** 2021-11-08

**Authors:** Ishak San, Emin Gemcioglu, Salih Baser, Nuray Yilmaz Cakmak, Abdulsamet Erden, Seval Izdes, Ramis Catalbas, Mehmet Davutoglu, Berkan Karabuga, Ihsan Ates

**Affiliations:** 1 Department of Emergency Medicine, University of Health Sciences, Ankara, Turkey; 2 Department of Internal Medicine, Ankara City Hospital, 06100 Ankara, Turkey; 3 Department of Internal Medicine, Yıldırım Beyazıt University Ankara City Hospital, Ankara, Turkey; 4 Department of Rheumatology, Ankara City Hospital, Ankara, Turkey; 5 Department of Intensive Care Unit, Yıldırım Beyazıt University, Ankara City Hospital, Ankara, Turkey

**Keywords:** Immunology, Microbiology, Biomarkers, Medical research, Risk factors

## Abstract

In this study, we compare the predictive value of clinical scoring systems that are already in use in patients with Coronavirus disease 2019 (COVID-19), including the Brescia-COVID Respiratory Severity Scale (BCRSS), Quick SOFA (qSOFA), Sequential Organ Failure Assessment (SOFA), Multilobular infiltration, hypo-Lymphocytosis, Bacterial coinfection, Smoking history, hyper-Tension, and Age (MuLBSTA) and scoring system for reactive hemophagocytic syndrome (HScore), for determining the severity of the disease. Our aim in this study is to determine which scoring system is most useful in determining disease severity and to guide clinicians. We classified the patients into two groups according to the stage of the disease (severe and non-severe) and adopted interim guidance of the World Health Organization. Severe cases were divided into a group of surviving patients and a deceased group according to the prognosis. According to admission values, the BCRSS, qSOFA, SOFA, MuLBSTA, and HScore were evaluated at admission using the worst parameters available in the first 24 h. Of the 417 patients included in our study, 46 (11%) were in the severe group, while 371 (89%) were in the non-severe group. Of these 417 patients, 230 (55.2%) were men. The median (IQR) age of all patients was 44 (25) years. In multivariate logistic regression analyses, BRCSS in the highest tertile (HR 6.1, 95% CI 2.105–17.674, p = 0.001) was determined as an independent predictor of severe disease in cases of COVID-19. In multivariate analyses, qSOFA was also found to be an independent predictor of severe COVID-19 (HR 4.757, 95% CI 1.438–15.730, p = 0.011). The area under the curve (AUC) of the BRCSS, qSOFA, SOFA, MuLBSTA, and HScore was 0.977, 0.961, 0.958, 0.860, and 0.698, respectively. Calculation of the BRCSS and qSOFA at the time of hospital admission can predict critical clinical outcomes in patients with COVID-19, and their predictive value is superior to that of HScore, MuLBSTA, and SOFA. Our prediction is that early interventions for high-risk patients, with early identification of high-risk group using BRCSS and qSOFA, may improve clinical outcomes in COVID-19.

## Introduction

COVID-19, caused by severe acute respiratory syndrome coronavirus 2 (SARS-CoV-2), is a pandemic infectious disease that causes morbidity and mortality. The prognosis of the disease may range from complete well-being to severe acute respiratory distress syndrome or death. Clinicians use different scoring systems to predict the prognosis of the disease, but there is no proven prognostic scoring system yet. The fact that many clinical, hematological, and biochemical parameters change during the inflammation process of COVID-19 suggests that it is possible to form an idea about the prognosis of the disease with scoring systems. Rapid and accurate clinical identification of patients infected with SARS-CoV-2 who are at risk of poor outcomes is a priority.

The Sequential Organ Failure Assessment (SOFA) score has value as an important diagnostic marker for sepsis and septic shock^1^. The SOFA score was originally determined to focus especially on organ failure and morbidity in order to show morbidity severity^2^. From the patient’s baseline risk, if the SOFA score is 2 or higher, the mortality risk is approximately 10% in relation to the general hospital population with presumed infection. We can also assume that risk of death is increased by 2 to 25 times compared to patients with a SOFA score of less than 2^1,3^. A SOFA score of ≥ 3 signifies organ failure for the relevant system^4^. For patients who have higher SOFA scores and lymphocytopenia on admission, there is a greater risk of developing severe COVID-19 disease^2^.

The quickSOFA (qSOFA) system has also been developed as a bedside clinical scoring system to classify patients clinically according to the severity of sepsis. If the qSOFA score is 2 or higher, it may be predictive of poor prognosis^1^. In a study where qSOFA was calculated in the emergency department for patients with suspected infection, the mortality rate was 3% for patients with qSOFA scores of ≤ 1 compared to 24% for patients with qSOFA scores of ≥ 2^5^. However, Ferreira et al. reported that qSOFA was not significant for identifying a COVID-19 patient with poor outcomes typical of sepsis^6^.

The MuLBSTA score (“Multilobular infiltration, hypo-Lymphocytosis, Bacterial coinfection, Smoking history, hyper-Tension, and Age”) can be used as an early mortality predictor among patients with viral pneumonia, and it has been suggested that it may play a role in predicting early mortality for COVID-19 patients^7,8^. In one study, the neutrophil-to-lymphocyte ratio (NLR) was found to be significantly and positively correlated with MuLBSTA scores in patients with COVID-19^9^.

Fardet et al. developed the HScore to help clinicians in the differential diagnosis of reactive hemophagocytic syndrome (RHS)^10^, which is usually known as macrophage activation syndrome (MAS) when it is secondary to a rheumatic disease^11^. Recently, the HScore has been suggested to evaluate critically ill COVID-19 patients to be able to start immunosuppression at the right time, because cytokine assays are expensive and not always available in general practice^12^. On the other hand, some authors have said that it may not be appropriate to use the HScore to guide the use of immunomodulatory therapy^13,14^.

During the management of COVID-19 patients, intensivists have had limited guidance on management. Researchers created the Brescia-COVID Respiratory Severity Scale (BRCSS) to help clinicians distinguish the severe form of COVID-19 from non-severe cases by sharing experiences between physicians of different specialties. The BCRSS score is suggested to be ≥ 3 for tocilizumab treatment^15,16^.

Since intensive care units are costly units with limited numbers of beds, proper use of resources is required. The use of scoring systems is required in intensive care units to reduce costs, use resources effectively, and guide clinical decisions and practices^17–19^. Furthermore, it is crucial to distinguish severe from non-severe COVID-19 at admission. To date, there is no antiviral treatment proven to be effective for COVID-19. That is why it is important to recognize and closely monitor high-risk patients to perform the necessary interventions on time. In this study, we compare the predictive values of the clinical scoring systems that are already in use in patients with COVID-19, namely the BCRSS, qSOFA, SOFA, MuLBSTA, and HScore, for determining the severity of the disease. Our aim in this study is to determine which scoring systems are most useful in determining disease severity and to guide clinicians.

## Materials and methods

In this study, 417 patients older than 18 years of age who were hospitalized in the internal diseases and infectious diseases wards of Ankara City Hospital due to COVID-19 were evaluated retrospectively. Patients younger than 18 years old, patients with active malignancy, and pregnant women were excluded from the study. Ethical approval of the study was obtained from the Ethics Committee of Ankara City Hospital (Date: 24/02/2021, Number: E2-21-140). The age, gender, comorbidities, and medications of the patients were recorded, as well as fever, respiratory rate, SpO_2_, d-dimer, fibrinogen, complete blood count, biochemical parameters, CRP, sedimentation rate, and thorax CT findings at admission to the emergency department. Demographic, clinical, laboratory, imaging examination, treatment, and outcome data were collected using a standardized case-report form. All data were checked by 2 physicians (EG and IA), and then a third researcher (SB) determined any differences in interpretation between the 2 primary reviewers.

All of the patients included in this study were tested for influenza A virus, influenza B virus, respiratory syncytial virus, and parainfluenza virus, and these infections were excluded by serological test. Nasal and/or pharyngeal swab specimens were collected from all patients, and reverse transcriptase-polymerase chain reaction assays were performed. In our tertiary medical facility, the patients received the diagnosis either by positive polymerase chain reaction (PCR) for COVID-19 or by fulfilling any 4 of 5 clinical criteria including fever, respiratory symptoms, history, compatible chest imaging findings, and decreased lymphocyte count^20,21^.

In this study, we classified the patients into two groups according to the stage of the disease (severe and non-severe) and adopted interim guidance of the World Health Organization^22^. Severe cases were divided into a group of surviving patients and a group of deceased patients according to their final prognosis.

Hospitalization, treatment, management, and discharge of the patients were decided according to the guidelines of the Turkish Ministry of Health^23^.

### Scoring systems

Five scores were included in this analysis to understand the relation between the severity groups of the COVID-19 patients, including the BCRSS, qSOFA, SOFA, MuLBSTA, and HScore. According to admission values, the BCRSS, qSOFA, SOFA, MuLBSTA, and HScore were evaluated at admission using the worst parameters available in the first 24 h^1–3,5,7,8,10,15,16^.

In our study, it was aimed to calculate the sensitivity and specificity values according to the cut-off values in the literature, as well as finding the best cut-off value of the scores. The cut-off values in the literature were used for these calculations, and the BCRSS, qSOFA, SOFA, MuLBSTA, and HScore values were 3, ≥ 2, ≥ 2, > 12, and > 169 in the calculations, respectively^1–3,5,7,8,10,15,16^.

### SOFA score

The Sepsis-Related Organ Failure Assessment score was developed by the European Society of Intensive Care Medicine to define the degree of organ failure due to sepsis^1–3^. However, since its validity was determined in patients with non-sepsis organ dysfunction, it was later renamed “Sequential Organ Failure Assessment” (SOFA). Six organ systems (respiratory, cardiovascular, central nervous, renal, coagulation, and liver systems) are scored between 1 and 4 points, with a total score between 6 and 24^1–3^. The score is based on the worst value in the last 24 h. If there is a value that cannot be measured, scoring is performed according to the closest measurement value.

### qSOFA

The Sepsis-3 definitions have facilitated earlier identification of patients at risk of developing sepsis for treatment^5^. QuickSOFA (qSOFA) is a bedside clinical score to clinically categorize a septic patient. In out-of-hospital, emergency department, or general hospital ward settings, adult patients with suspected infection can be rapidly identified as being more likely to have poor outcomes typical of sepsis if they have at least 2 of the following clinical criteria of qSOFA: respiratory rate of 22/min or greater, altered mentation, or systolic blood pressure of ≤ 100 mmHg. This definition was later confirmed in the emergency department for patients with suspected infection^5^.

### MuLBSTA

The MuLBSTA score is a scoring system developed to predict 90-day mortality in viral pneumonia patients with multilobular infiltration, lymphopenia, bacterial coinfection, smoking history, hypertension, and age of ≥ 60 years^7,8^. All parameters defined in the MuLBSTA score are clinically easy to obtain, and it is recommended that all examinations be performed on admission. The MuLBSTA score was developed as a marker that shows the risk in the clinical prediction of patients specifically diagnosed with viral pneumonia^8^. The risk categories and death rates for each grade are suggested as follows: MuLBSTA 0–11, low risk, mortality of 5.07%; and MuLBSTA 12–22, high risk, mortality of 33.92%^8^.

### HScore

Nine variables are used for the HScore as follows: three clinical variables (high fever, organomegaly, underlying immunosuppression), five biochemical variables (triglycerides, ferritin, serum transaminases, fibrinogen, presence of cytopenia), and one cytological variable (findings of hemophagocytosis in the bone marrow)^10^. The best cut-off value in hemophagocytic syndrome (HPS) for the HScore was 169, and it exactly classified 90% of patients with 93% sensitivity and 86% specificity^10^.

### BRCSS

The Brescia-COVID Respiratory Severity Scale (BRCSS) was created by sharing experiences among physicians of different specialties^15,16^. Since the beginning of the COVID-19 pandemic in Lombardy, a daily multidisciplinary meeting has been held to coordinate patient care and transfer between units. Participants of these meetings have included intensive care, infectious diseases, chest diseases, immunology, rheumatology, and internal medicine specialists. The BRCSS uses clinical criteria to rank non-intubated patients. It assigns patients a score of 0–3 based on 4 test criteria: (1) dyspnea or staccato speech, defined as being unable to count rapidly up to 20 after a deep breath, at rest, or during minimal activity, such as sitting up in bed, standing, talking, swallowing, or coughing; (2) respiratory rate of > 22 breaths/min; (3) PaO_2_ of < 65 mmHg or SpO_2_ of < 90% with supplemental oxygen; and (4) significant worsening of chest radiography. In intubated patients, PaO_2_/FiO_2_ below 150 mmHg determines whether the score is 5 or above, and the use of adjunctive therapies including prone positioning and neuromuscular blockade agents further increases the score^15,16^. The BRCSS may be useful for practicing clinicians to gauge the clinical improvement or worsening of patients infected with SARS-CoV-19. It may be used in other countries, as well^15^.

### Statistical analysis

The data were analyzed using SPSS for Windows version 25.0 (IBM Corp., Armonk, NY, USA) and MedCalc 15.8 (Franz Faul, Universitat Kiel, Germany). While frequency, percentage, mean, standard deviation, median, and IQR were used as descriptive statistical methods, the chi-square (χ^2^) test was used to compare qualitative data. The consistency of the data with normal distribution was evaluated by the Kolmogorov–Smirnov and Shapiro–Wilk tests. As a result, the median was used because not all of the data were homogeneously distributed. The Mann–Whitney U test was used to compare the data not consistent with normal distribution. While the receiver operating characteristic (ROC) curve method was used to determine the discrimination of the variables, binary logistic regression was used to determine the risk rates. The variables for which the unadjusted p-value was < 0.10 in logistic regression analysis were identified as potential risk markers and included in the full model. We reduced the model by using backward conditional elimination multivariate logistic regression analyses and eliminated potential risk markers by using likelihood ratio tests. The clinically valuable ones of the correlations were added.

The statistical significance level was considered as p < 0.05.

### Ethics committee approval

Ankara City Hospital No.2 Clinical Research Ethics Committee approval was received for this study at 24.02.2021, Approval number: E2-21-140.

### Helsinki declaration

The authors declared that this study is in accordance with the Helsinki Declaration.

### Informed consent

Informed consent from patients and from legal guardians of deceased patients was obtained for the study.

## Results

Of the 417 patients included in our study, 46 (11%) were in the severe group, while 371 (89%) were in the non-severe group. Of these 417 patients, 230 (55.2%) were men. The median (IQR) age of all patients was 44 (25) years (Table 1).

**Table 1:** Evaluation of the mild and severe patient groups according to clinical status, demographics, past-history, laboratory parameters and scores.

Characteristics or Findings	All Patients n:417	Severe n:46	Non-Severe n:371	p value
Male sex—no. (%)	230 (55.2)	28 (60.9)	202 (54.4)	0.504
Median age, (IQR) years	44 (25)	67.5 (13.25)	42 (23)	< 0.0001
Cough—no. (%)	241 (57.8)	28 (60.9)	213 (57.4)	0.772
Fever—no. (%)	189 (45.3)	28 (60.9)	161 (43.4)	0.037
Dyspnea—no. (%)	93 (22.3)	14 (30.4)	79 (21.3)	0.224
Headache—no. (%)	41 (9.8)	4 (8.7)	37 (10)	1.000
Myalgia—no. (%)	115 (27.6)	13 (28.3)	102 (27.5)	1.000
Diarrhea—no. (%)	25 (6.0)	2 (4.3)	23 (6.2)	1.000
Back pain—no. (%)	2 (0.5)	0 (0)	2 (0.5)	1.000
Anosmia—no. (%)	28 (6.7)	3 (6.5)	25 (6.7)	1.000
Ageusia—no. (%)	24 (5.8)	2 (4.3)	22 (5.9)	1.000
Abdominal pain—no. (%)	2 (0.5)	0 (0)	2 (0.5)	1.000
Arthralgia—no. (%)	19 (4.6)	0 (0)	19 (5.1)	0.249
Smoking [Acute and Quit-smoker]—no. (%)	21 (5)	4 (8.7)	17 (4.6)	0.074
Any Comorbidity—no. (%)	127 (30.5)	28 (60.9)	99 (26.7)	< 0.0001
Hypertension—no. (%)	79 (18.9)	20 (43.5)	59 (15.9)	< 0.0001
Diabetes—no. (%)	54 (12.9)	9 (19.6)	45 (12.1)	0.236
Asthma—no. (%)	16 (3.8)	2 (4.3)	14 (3.8)	0.693
Obesity—no. (%)	4 (1)	0 (0)	4 (1.1)	1.000
CHD—no. (%)	32 (7.7)	11 (23.9)	21 (5.7)	< 0.0001
Renal disease—no. (%)	6 (1.4)	0 (0)	6 (1.6)	1.000
Creatinin (mg/dL)	0.81 (0.27)	0.88 (0.41)	0.8 (0.27)	0.194
AST (U/L)	23 (16)	32.5 (48.25)	22 (16)	< 0.0001
ALT (U/L)	26 (21)	29.5 (43.25)	25 (20)	0.005
LDH (U/L)	218 (88.5)	299 (216.5)	215 (77)	< 0.0001
CRP (mg/L)	0.01 (0.02)	0.04 (0.11)	0.01 (0.02)	< 0.0001
ESR (mm/h)	18 (31)	50.5 (50.25)	16.5 (25)	< 0.0001
Ferritin (μg/L)	122 (227.5)	282 (507)	110 (210.9)	< 0.0001
WBC (10^9^/L)	5.2 (2.61)	6.52 (4)	5.13 (2.38)	< 0.0001
Lymphocyte (10^9^/L)	1.28 (0.73)	0.98 (0.48)	1.30 (0.71)	< 0.0001
Platelet (10^9^/L)	213 (97)	193.5 (122.25)	217 (95)	0.635
Hemoglobulin (g/dL)	13.8 (2.35)	12.25 (2.88)	13.9 (2.2)	< 0.0001
SOFA	0 (1)	3 (3)	0 (1)	< 0.0001
qSOFA	0 (0)	2 (2)	0 (0)	< 0.0001
MuLBSTA	5 (6)	11 (6,5)	5 (6)	< 0.0001
HScore	47 (52)	75 (47.25)	44 (48)	< 0.0001
BRCSS	0 (1)	3 (4)	0 (1)	< 0.0001

All laboratory parameters have been calculated as median (IQR).

*ESR* Erythrocyte sedimentation rate, *CRP* C-reactive protein, *ICU* intensive care unit, *CHD* coronary heart disease, *WBC* white blood cell count, *AST* serum aspartate aminotransferase, *ALT* serum alanine aminotransferase, *LDH* lactate dehydrogenase, *SOFA* sequential organ failure assessment score, *qSOFA* quick sequential organ failure assessment score, *MuLBSTA* multilobularinfiltration, hypo-lymphocytosis, bacterial coinfection, smoking history, hyper-tension and age score, *HScore* hemophagocytosis score, *BRCSS* Brescia respiratory covid severity scale.

Demographic data, clinical data, laboratory parameters, and scores of patients are compared in terms of severe and non-severe cases in Table 1. Median age (IQR) in patients with severe disease was higher than that of non-severe patients [67.5 (13.25) vs. 42 (23), p < 0.0001]. Those with severe disease had more comorbidities than those with non-severe disease [28 (60.9%) vs. 99 (26.7%), p < 0.0001]. Among the comorbidities, the frequency of hypertension was 20 (43.5%) in the severe disease group and 59 (15.9%) in the non-severe group (p < 0.0001). The frequency of coronary heart disease was 11 (23.9%) in the severe disease group and 21 (5.7%) in the non-severe group (p < 0.0001). While the frequency of fever was 28 (60.9%) in patients with severe disease, it was 161 (43.4%) in non-severe cases.

Serum aspartate aminotransferase, serum alanine aminotransferase, lactate dehydrogenase, C-reactive protein, sedimentation rate, ferritin concentration, and white blood cell count values were significantly higher in the severe patient group compared to the non-severe group (all p < 0.005) (Table 1). Hemoglobin and lymphocyte values were significantly lower in the severe patient group than in the non-severe patient group (p < 0.0001 for both).

The median (IQR) SOFA score was 3 (3) in the severe patient group and 0 (1) in the non-severe patient group (p < 0.0001). The median (IQR) qSOFA score was 2 (2) in the severe patient group and 0 (0) in the non-severe patient group (p < 0.0001). The median (IQR) MuLBSTA score was 11 (6.5) in the severe patient group and 5 (6) in the non-severe patient group (p < 0.0001). The median (IQR) HScore was 75 (47.25) in the severe patient group and 44 (48) in the non-severe patient group (p < 0.0001). While the median (IQR) BRCSS was 3 (4) in the severe patient group, it was 0 (1) in the non-severe patient group (p < 0.0001).

Demographic data, clinical data, laboratory parameters, and scores of patients compared in terms of survival versus death are shown in Table 2. There was no significant difference between the median (IQR) age of severe-surviving patients and those of severe-deceased patients [66 (19) vs. 71 (12), p = 0.685]. Comorbidities were higher in severe-surviving patients than in severe-deceased patients [13 (86.7%) vs. 15 (48.4%), p < 0.03]. Among the comorbidities, the frequency of hypertension was 20 (43.5%) in the severe disease group and 59 (15.9%) in the non-severe group (p < 0.0001).

**Table 2:** Evaluation of the clinical status, demographics, past-history, laboratory parameters and scores between alive and dead severe patients at the time of admission.

	Severe Covid-19		p
	Severe-alive n:31	Severe-dead n:15	
Male sex—no. (%)	20 (64.5)	8 (53.3)	0.685
Median age (IQR) years	66 (19)	71 (12)	0.136
Any Comorbidity—no. (%)	15 (48.4)	13 (86.7)	0.030
Creatinin (mg/dL)	0.79 (0.27)	1.10 (1.69)	0.016
AST (U/L)	28 (20)	77 (86)	0.002
ALT (U/L)	29 (37)	45 (138)	0.038
LDH (U/L)	256 (121)	469 (390)	0.002
CRP (mg/L)	0.02 (0.09)	0.09 (0.15)	0.009
ESR (mm/h)	53 (56)	45 (46.75)	0.470
Ferritin concentration (μg/L)	228 (355)	1139 (1341.25)	0.002
WBC, (10^9^/L)	6.1 (3)	8.14 (10.14)	0.073
Lymphocyte (10^9^/L)	1.01 (0.53)	0.75 (0.59)	0.042
Platelet (10^9^/L)	236 (189)	180 (84)	0.017
Hemoglobulin (g/dL)	12.3 (2.9)	11.6 (4)	0.114
SOFA	2 (1)	8 (7)	< 0.0001
qSOFA	1 (1)	3 (0)	< 0.0001
MuLBSTA	9 (6)	15 (5)	0.002
HScore	74 (45)	96 (77)	0.011
BRCSS	3 (0)	7 (1)	< 0.0001

All laboratory parameters have been calculated as median (IQR).

*ESR* Erythrocyte sedimentation rate, *CRP* C-reactive protein, *ICU* Intensive care unit, *CHD* Coronary heart disease, *WBC* White blood cell count, *AST* Serum aspartate aminotransferase, *ALT* Serum alanine aminotransferase, *LDH*; Lactate dehydrogenase, *SOFA* sequential organ failure assessment score, *qSOFA* quick sequential organ failure assessment score, *MuLBSTA* multilobularinfiltration, hypo-lymphocytosis, bacterial coinfection, smoking history, hyper-tension and age score, *HSCORE* hemophagocytosis score, *BRCSS* Brescia respiratory covid severity scale.

Serum aspartate aminotransferase, serum alanine aminotransferase, lactate dehydrogenase, C-reactive protein, sedimentation rate, ferritin concentration, and white blood cell count values were significantly higher in the severe-deceased patient group compared to the severe-surviving group (all p < 0.005) (Table 2). Platelet and lymphocyte values were significantly lower in the severe-deceased patient group than in the severe-surviving patient group (p < 0.005 for both).

The median (IQR) SOFA score was 8 (7) in the severe-deceased patient group and 2 (1) in the severe-surviving patient group (p < 0.0001). The median (IQR) qSOFA score was 3 (0) in the severe-deceased patient group and 1 (1) in the severe-surviving patient group (p < 0.0001). The median (IQR) MuLBSTA score was 15 (5) in the severe-deceased patient group and 9 (6) in the severe-surviving patient group (p < 0.002). The median (IQR) HScore was 96 (77) in the severe-deceased patient group and 74 (45) in the severe-surviving patient group (p < 0.011). While the median (IQR) BRCSS was 7 (1) in the severe-deceased patient group, it was 3 (0) in the severe-surviving patient group (p < 0.0001).

A multivariate logistic regression model for severe disease consisting of the variables of age, any comorbidity, SOFA, qSOFA, MuLBSTA, HScore, and BRCSS is shown in Table 3. In the multivariate logistic regression analyses, BRCSS in the highest tertile (HR 6.1, 95% CI 2.105–17.674, p = 0.001) was determined as an independent predictor of severe COVID-19. In multivariate analyses, qSOFA was also found to be an independent predictor of severe disease in COVID-19 cases (HR 4.757, 95% CI 1.438–15.730, p = 0.011).

**Table 3:** Evaluation of the parameters of all patients according to the severity of disease with multivariate logistic regression analyses at the time of admission.

Parameters	OR	95% CI	p value
Age, years	1.013	0.968–1.059	0.589
Any Comorbidity	1.524	0.409–5.686	1.524
SOFA	1.180	0.747–1.865	0.478
qSOFA	4.757	1.438–15.730	0.011
MuLBSTA	1.121	0.928–1.354	0.236
HScore	1.004	0.985–1.023	0.700
BRCSS	6.100	2.105–17.674	0.001

*SOFA* Sequential organ failure assessment score, *qSOFA* quick sequential organ failure assessment score, *MuLBSTA* multilobularinfiltration, hypo-lymphocytosis, bacterial coinfection, smoking history, hyper-tension and age score, *HScore* hemophagocytosis score, *BRCSS* Brescia respiratory covid severity scale.

**Figure 1:**
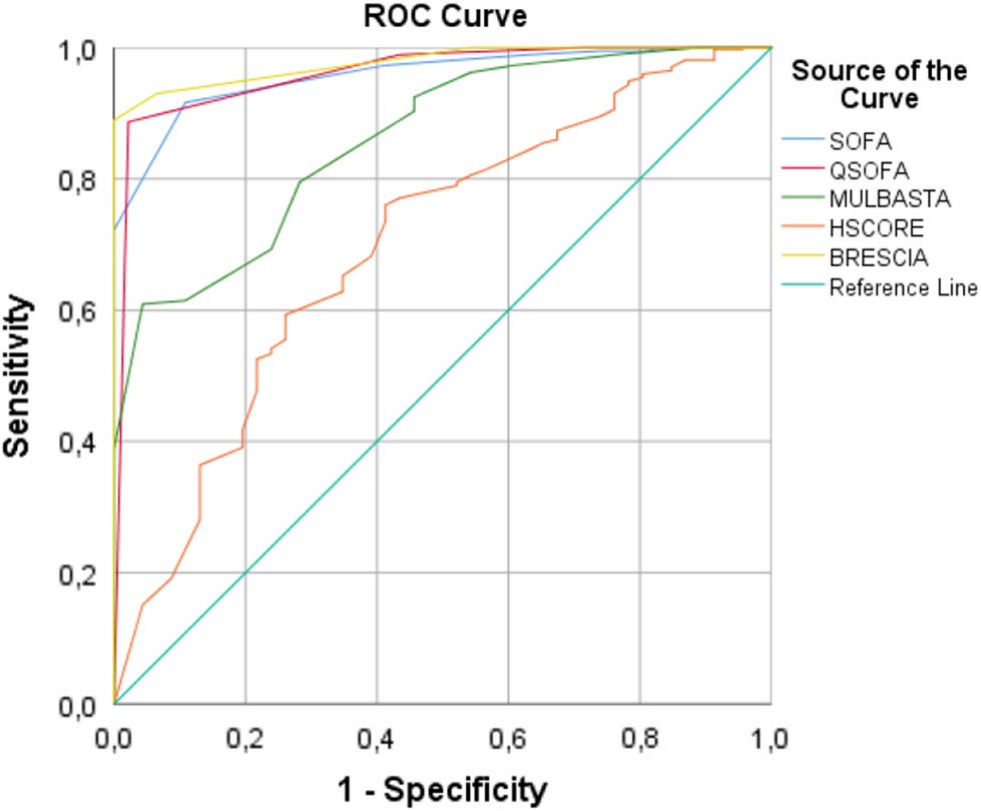
Receiver operating characteristic analysis of BRCSS, qSOFA, SOFA, MuLBSTA and HScore for severe disease of Covid-19.

The values of these five scores in all patients with severe cases of COVID-19 were calculated, and the predicted values of these scores were compared in ROC analysis (Fig. 1). In Table 4, the area under the curve (AUC) of the BRCSS, qSOFA, SOFA, MuLBSTA, and HScore is seen to be 0.977, 0.961, 0.958, 0.860, and 0.698, respectively. All of these scores could be used as potential diagnostic biomarkers for subsequent analysis because their AUC values are higher than 0.50. The optimal cut-off values were > 1, ≥ 1, > 1, > 5, and > 72 for BRCSS, qSOFA, SOFA, MuLBSTA, and HScore, respectively. In Table 5, we re-evaluate the sensitivity and specificity of the scores according to the cut-off values given in the literature. When evaluated in this way, the specificity of the HScore was 100% and its sensitivity was 0%. The cut-off we found for the SOFA score was the same as the cut-off value in the literature. While we found the cut-off of the BRCSS as ≥ 3, the same as in the literature, the sensitivity was 93.48% and the specificity was 92.99%.

**Table 4:** The AUC and optimal thresholds of each independent risk or protection factors for score system of all patients according to the severity of disease.

Indicators	AUC	p value	Optimal threshold	Sensitivity	Specificity	95% CI	Youden index
SOFA	0.958	< 0.0001	> 1	89.13	91.64	0.934–0.975	0.808
qSOFA	0.961	< 0.0001	≥ 1	97.83	88.68	0.937–0.977	0.865
MuLBSTA	0.860	< 0.0001	> 5	95.65	60.92	0.823–0.892	0.566
HScore	0.698	< 0.0001	> 72	58.7	76.01	0.651–0.741	0.347
BRCSS	0.977	< 0.0001	> 1	100	88.95	0.958–0.989	0.890

*SOFA* Sequential organ failure assessment score, *qSOFA* quick sequential organ failure assessment score, *MuLBSTA* multilobularinfiltration, hypo-lymphocytosis, bacterial coinfection, smoking history, hyper-tension and age score, *HScore* hemophagocytosis score, *BRCSS* Brescia respiratory covid severity scale.

**Table 5:** Evaluation of the sensitivity and specificity of the scoring systems according to the cut-off values in literature.

Indicators	AUC	p value	Optimal threshold	Sensitivity	Specificity	95% CI	Youden index
SOFA	0.958	< 0.0001	≥ 2	89.13	91.64	0.934–0.975	0.808
qSOFA	0.961	< 0.0001	≥ 2	56.52	98.92	0.937–0.977	0.865
MuLBSTA	0.860	< 0.0001	≥ 12	45.65	96.23	0.823–0.892	0.566
HScore	0.698	< 0.0001	> 169	0	100	0.651–0.741	0.347
BRCSS	0.977	< 0.0001	≥ 3	93.48	92.99	0.958–0.989	0.890

*SOFA* Sequential organ failure assessment score, *qSOFA* quick sequential organ failure assessment score, *MuLBSTA* multilobularinfiltration, hypo-lymphocytosis, bacterial coinfection, smoking history, hyper-tension and age score, *HScore* hemophagocytosis score, *BRCSS* Brescia respiratory covid severity scale.

## Discussion

As far as we know, our work is the first in the literature that compares the BRCSS, qSOFA, SOFA, MuLBSTA, and HScore scoring systems for COVID-19 patients in a single study. We found that SOFA, qSOFA, MuLBSTA, HScore, and BRCSS scores were all significantly higher in the severe patient group compared to the non-severe patient group.

The SOFA score has great value to show the severity of multiple organ dysfunction^24^. In the study of Yao et al., it was reported that lymphopenia and high SOFA score at the time of admission to the hospital helped clinicians identify patients with high risk of severe Covid-19 infection^2^. In the same study, conducted with 108 patients, the median SOFA score was found to be 2 in the severe patient group and 1 in the non-severe patient group, while the SOFA score was found to be significantly higher in severe-deceased patients compared to severe-surviving patients. Similarly, in our study, the SOFA score of severe-deceased patients was significantly higher than the SOFA score of severe-surviving patients. Similar to our study, in a study conducted by Zhou et al., the SOFA score was found to be 4.5 in severe-deceased patients, while it was found to be 1 in severe-surviving patients^13^. In a study conducted by Wang et al. in Wuhan, a median SOFA score above 4 was found to be associated with mortality^25^. In our study, the median for this score was found to be 8 in severe-deceased patients. Another study reported that higher SOFA score on admission was associated with increased odds of severe COVID‑19 and was an independent risk factor for death (OR 2.45, 95% CI 1.302–4.608, p = 0.005 and OR 2.402, 95% CI 1.313–4.395, p = 0.004)^2^. Although higher SOFA score at admission was identified as an independent predictor for developing severe SARS‑CoV‑2 infection in some research, we could not detect SOFA as an independent predictor when we evaluated it along with other scores in multivariate analysis^2,25,26^.

Compared to the SOFA score, the qSOFA is a simpler and more useful criterion to indicate severity prediction for in-hospital mortality (AUC 0.81, 95% CI 0.80–0.82). It was also reported that qSOFA was statistically superior to SOFA or change in SOFA score in non-ICU patients^27^. In a study conducted by Jang et al., the qSOFA score was found to be significantly higher in critically ill patients, similar to our study^28^. Another study reported that the qSOFA scores of ventilated patients with COVID-19 were 1 or less in 27 patients (87%) and only 4 patients had a 2-point qSOFA, while none had 3 points. Therefore, in the study of Ferreira et al., the authors anticipated that the qSOFA was not appropriate to identify COVID-19 patients having poor outcomes typical of sepsis^6^. Another study about COVID-19 showed that the risk factors significantly associated with admission to the intensive care unit were a qSOFA score above 0 (OR 2.80, 95% CI 1.25–6.26, p = 0.012) upon multivariate analysis^29^. In our study, qSOFA was found to be an independent predictor of severe disease in COVID-19 cases upon multivariate analyses (HR 4.757, 95% CI 1.438–15.730). The qSOFA cut-off value of ≥ 1 had the highest AUC (0.961, 95% CI 0.937–0.977) after the BRCSS in predicting severe disease.

Chen et al. showed that the MuLBSTA score has a strong predictive ability for 90-day mortality in COVID-19^7^. In a study by Xiao et al., the MuLBSTA score was found to be more significant in determining the severity of COVID-19 infection compared to other scores, and it was stated that it could play a role in determining mortality in the early stages of the disease^30^. In our study, the MuLBSTA score in severe cases was found to be significantly higher than those of non-severe patients. In addition, in our study, the median MuLBSTA score was found to be 15 in severe-deceased patients, while it was 9 in severe-surviving patients (p = 0.002). In a study in which patients with COVID-19 were evaluated according to MuLBSTA scores, the score was 6.73 ± 2.29 in the non-ARDS group and 8.94 ± 2.69 in the ARDS group (p < 0.001)^31^. In that study, for the ROC analysis of the MuLBSTA score to predict ARDS, when the MuLBSTA value was > 8.00, the AUC was 0.730 (0.661–0.792, p < 0.0001)^31^. In our study, for the ROC analysis of the MuLBSTA score to predict severe disease, when the MuLBSTA value was > 5, the AUC was 0.860. In parallel with our study, the studies in the literature show that the MuLBSTA score is a reliable scoring system to show the severity of COVID-19 infection, but further evaluation is required for this scoring system in cases of COVID-19 pneumonia. When the cut-off value of the MuLBSTA score in the literature was evaluated by ROC analysis, its sensitivity was found to be 45.65%, while the specificity was found as 96.23%. However, we did not find this score to be an independent predictor for severe disease in multivariate analysis.

In a study by Bhattacharjee et al., it was stated that the HScore can be used to determine the time to initiate immunotherapy in patients with severe COVID-19 infection^6,32^. A recent study showed that high HScore values were more indicative of MAS patients than severe COVID-19 patients (201.9 ± 15.3 vs. 88.8 ± 48.3, p < 0.0001). The same study results showed that all MAS patients met the diagnostic cut-off value of the HScore (> 169), but only 10% of severe COVID-19 patients did. Thus, further investigations are required to assess its effectiveness for severe COVID-19 cases^33^. Another study showed that the HScore could not predict the clinical severity of COVID-19 patients characterized by hyperinflammation-mediated respiratory failure, and it also found that it was not effective in predicting admission to the intensive care unit^10^. Similarly, according to our study, the HScore was insufficient in detecting cases in the early period. On the other hand, the presence of underlying disease is an important factor for cut-off values recommended for the HScore^34^. Although it is more successfully used in the diagnosis of hemophagocytic lymphohistiocytosis, it has several limitations in the evaluation of COVID-19 patients. Hyperferritinemia is an important marker for secondary hemophagocytic lymphohistiocytosis (sHLH), but although increased ferritin is observed in patients with COVID-19, the HScore threshold of 2000 ng/mL is seen in late stages of the disease and may delay the treatment given to these patients^35^. In our study, the HScore values of patients with severe disease were found to be significantly higher compared to those of non-severe patients. In addition, the HScore values of severe-deceased patients were found to be higher than those of severe-surviving patients. However, these values are far from the cut-off values used for sHLH in the literature, and this may be due to the limitations mentioned above. In addition, it is not easy to perform invasive procedures such as bone marrow aspiration during pandemic processes. Because of that, bone marrow aspiration could be a disadvantage of the HScore. As a result, the limitations of the HScore are the need for both bone marrow aspiration and calculations^36^. If the HScore is to be used in COVID-19, a new cut-off value should be determined without bone marrow aspiration.

The BCRSS, on the other hand, is a scale used to determine the respiratory severity of COVID-19 pneumonia, showing the patient’s need for oxygen and mechanical ventilation, and it yields a score between 0 and 8. As the score increases, the respiratory severity of COVID-19 pneumonia and the patient’s need for oxygen increase^16,37,38^. The BCRSS classifies the severity of patients according to the factors of oxygen supplementation and ventilatory support requirements, and it guides the clinician in decisions about further therapeutic investments like antiviral and/or anti-inflammatory drugs. In a study of 236 patients by Moreno-Perez et al., low comorbidity assessed by the BCRSS and early response to tocilizumab treatment were associated with survival and were evaluated as a guide in the follow-up of COVID-19 patients treated with tocilizumab^38^. In parallel with these studies, in our study, the median BCRSS was found to be 7 in severe-deceased patients, while it was 3 in severe-surviving patients. In our study, BCRSS was found to be an independent predictor of severe disease in cases of COVID-19 based on multivariate analyses (HR 6.100, 95% CI 2.105–17.674). The BRCSS cut-off value of > 1 had the highest AUC value (0.977, 95% CI 0.958–0.989) compared to other scores in predicting severe disease. In a recent article in which 17 patients who were given anakinra treatment were evaluated retrospectively, it was found that the rate of patients with BCRSS scores of 3 or above was 88.2%^39^. The BCRSS determines the clinical summary of the status of the patients in a simple way and helps clinicians easily compare among patients. In our opinion, the reason why the BRCSS recognizes severe disease better in the early period is that the creators of this score included intensive care, infectious diseases, chest diseases, immunology, rheumatology, and internal medicine specialists^15,16^. Furthermore, the BCRSS uses patients’ examinational status according to the degree of respiratory supply (noninvasive ventilation, intubation, prone positioning) to recommend treatment modalities (anakinra, tocilizumab).

Our study had some limitations. First of all, this study was conducted in a single center and was a retrospective study. For validation, multiple neutral prospective studies need to be done. Secondly, this study included only five scoring systems to predict the severity of COVID-19 disease, but further studies may include additional scores. The third limitation of this study was that all the markers and measurements were evaluated only one time, at admission; therefore, changes in those parameters could not be evaluated. Our final limitation is that the introduction and discussion sections of this article are somewhat long due to the fact that we have examined five scores together.

The advantages of our study are as follows: compared to other current studies, many scores in the literature were evaluated together in our study, and we have investigated which scores best recognize severe patients earlier at admission. Our study differs from others since both the number of parameters and the number of patients are higher.

Calculation of the BRCSS and qSOFA scores at the time of hospital admission can predict critical clinical outcomes in patients with COVID-19, and their predictive value is superior to that of the HScore, MuLBSTA, and SOFA. By early detection of the high-risk group using the BRCSS and qSOFA, early interventions for high-risk patients can improve clinical outcomes in COVID-19 patients. The reason for the low accuracy of the HScore and MuLBSTA in COVID-19 clinical outcome prediction of is the presence of many “silent hypoxemia” patients among severe cases. Even if they seem to be breathing comfortably, their measured oxygen saturation is low in pulse oximetry. Thus, the HScore and MuLBSTA have a disadvantage in the prediction of severe cases. On the other hand, the reason for high accuracy in clinical outcome prediction of COVID-19 by the BCRSS can be explained by the fact that it evaluates breathing, hypoxia, and oxygen requirements.

According to these scores, patients are evaluated in terms of triage and hospitalization is decided in the intensive care unit, the necessary interventions are done by predicting the medical results of the patients, the procedures are developed in the hospital, and the budget and resources are used effectively. The early recognition of patients at risk of developing severe disease allows an appropriate approach that would be started at the time of ICU admission, and this would help reduce mortality. Furthermore, early prognosis prediction would help alleviate the shortage of medical resources.

## Conclusion

The data collected for our study included the patients’ test results at first admission. In our study, the HScore, MuLBSTA, SOFA, qSOFA, and BRCSS scores were all significantly high in the group of patients with severe COVID-19. All parameters identified in the BRCSS and qSOFA systems are clinically easy to obtain, and all examinations are recommended to be done at admission to the hospital. The BRCSS and qSOFA may help clinicians communicate and determine their treatment plans in the early period of COVID-19. These prognostic markers can be used to prioritize patients requiring intensive care and aggressive management.

## Data Availability

The datasets used and/or analysed during the current study are available from the corresponding author on reasonable request.

## References

[1] Singer M (2016). The third international consensus definitions for sepsis and septic shock (Sepsis-3). JAMA.

[2] Yao Q (2020). A retrospective study of risk factors for severe acute respiratory syndrome coronavirus 2 infections in hospitalized adult patients. Pol. Arch. Intern. Med..

[3] Seymour CW (2016). Assessment of clinical criteria for sepsis: For the third international consensus definitions for sepsis and septic shock (Sepsis-3). JAMA.

[4] Królicki T (2020). Systemic inflammatory markers predict detrimental outcome of urosepsis in kidney transplant recipients. Transplant. Proc..

[5] Freund Y (2017). Prognostic accuracy of sepsis-3 criteria for in-hospital mortality among patients with suspected infection presenting to the emergency department. JAMA.

[6] Ferreira M (2020). Critically ill SARS-CoV-2-infected patients are not stratified as sepsis by the qSOFA. Ann. Intensive Care..

[7] Chen N (2020). Epidemiological and clinical characteristics of 99 cases of 2019 novel coronavirus pneumonia in Wuhan, China: A descriptive study. Lancet.

[8] Guo L (2019). Clinical features predicting mortality risk in patients with viral pneumonia: The MuLBSTA Score. Front. Microbiol..

[9] Liu J (2020). Neutrophil-to-lymphocyte ratio predicts critical illness patients with 2019 coronavirus disease in the early stage. J. Transl. Med..

[10] Stephan JL (1993). Macrophage activation syndrome and rheumatic disease in childhood: A report of four new cases. Clin. Exp. Rheumatol..

[11] Fardet L (2014). Development and validation of the HScore, a score for the diagnosis of reactive hemophagocytic syndrome. Arthritis Rheumatol..

[12] Mehta P (2020). COVID-19: Consider cytokine storm syndromes and immunosuppression. Lancet.

[13] Zhou F (2020). Clinical course and risk factors for mortality of adult inpatients with COVID-19 in Wuhan, China: A retrospective cohort study. Lancet.

[14] Wood H (2020). Secondary HLH is uncommon in severe COVID-19. Br. J. Haematol..

[15] Piva S (2020). Clinical presentation and initial management critically ill patients with severe acute respiratory syndrome coronavirus 2 (SARS-CoV-2) infection in Brescia, Italy. J. Crit. Care..

[16] Lombardy Section Italian Society Infectious and Tropical Diseases (2020). Vademecum for the treatment of people with COVID-19. Edition 2.0, 13 March 2020. Infez. Med..

[17] Vincent J-L, Moreno R (2010). Clinical review: Scoring system in the critically ill. Crit. Care.

[18] Strand K, Flaatten H (2008). Severity scoring in the ICU: Review. Acta Anaesthesiol. Scand..

[19] Keegan MT, Gajic O, Afessa B (2011). Severity of illness scoring systems in the intensive care unit. Crit. Care Med..

[20] Shen KL (2020). Updated diagnosis, treatment and prevention of COVID-19 in children: Experts’ consensus statement (condensed version of the second edition). World J. Pediatr..

[21] Tezcan ME, Doğan Gökçe G, Ozer RS (2020). Laboratory abnormalities related to prolonged hospitalization in COVID-19. Infect. Dis. (Lond)..

[22] WHO. Clinical management of severe acute respiratory infection when novel coronavirus (2019-nCoV) infection is suspected: Interim guidance. (2020). https://apps.who.int/iris/handle/10665/330854. Accessed 25 Jan 2020.

[23] Ministry TH. Turkish Health Ministry. Guidance To Covid-19 (SARS Cov2 Infection). https://hsgm.saglik.gov.tr/tr/covid-19-ingilizce-dokumanlar.html. Accessed 8 June 2020.

[24] Ferreira FL (2001). Serial evaluation of the SOFA score to predict outcome in critically ill patients. JAMA.

[25] Wang ZH, Shu C, Ran X, Xie CH, Zhang L (2020). Critically ill patients with coronavirus disease 2019 in a designated ICU: Clinical features and predictors for mortality. Risk Manag. Healthc. Policy..

[26] Liu J (2020). Clinical outcomes of COVID-19 in Wuhan, China: A large cohort study. Ann. Intensive Care..

[27] Innocenti F (2018). SOFA score in septic patients: Incremental prognostic value over age, comorbidities, and parameters of sepsis severity. Intern. Emerg. Med..

[28] Jang JG, Hur J, Hong KS, Lee W, Ahn JH (2020). Prognostic accuracy of the SIRS, qSOFA, and NEWS for early detection of clinical deterioration in SARS-CoV-2 infected patients. J. Korean Med. Sci..

[29] Almazeedi S (2020). Characteristics, risk factors and outcomes among the first consecutive 1096 patients diagnosed with COVID-19 in Kuwait. EClinicalMedicine..

[30] Xiao LS (2020). Development and validation of the HNC-LL score for predicting the severity of coronavirus disease 2019. EBioMedicine.

[31] Peng J (2020). Diagnostic value of peripheral hematologic markers for coronavirus disease 2019 (COVID-19): A multicenter, cross-sectional study. J. Clin. Lab. Anal.

[32] Bhattacharjee S, Banerjee M, Pal R (2020). COVID-19-associated hemophagocytic lymphohistiocytosis and coagulopathy: Targeting the duumvirate. Indian Pediatr..

[33] Ruscitti P (2020). Lung involvement in macrophage activation syndrome and severe COVID-19: Results from a cross-sectional study to assess clinical, laboratory and artificial intelligence-radiological differences. Ann. Rheum. Dis..

[34] Soy M, Atagündüz P, Atagündüz I, Sucak GT (2020). Hemophagocytic lymphohistiocytosis: A review inspired by the COVID-19 pandemic. Rheumatol. Int..

[35] Leverenz DL, Tarrant TK (2020). Is the HScore useful in COVID-19?. Lancet.

[36] Batu ED (2017). Assessment of the HScore for reactive haemophagocytic syndrome in patients with rheumatic diseases. Scand. J. Rheumatol..

[37] Toniati P (2020). Tocilizumab for the treatment of severe COVID-19 pneumonia with hyperinflammatory syndrome and acute respiratory failure: A single center study of 100 patients in Brescia, Italy. Autoimmun. Rev..

[38] Moreno-Pérez O (2020). Experience with tocilizumab in severe COVID-19 pneumonia after 80 days of follow-up: A retrospective cohort study. J. Autoimmun..

[39] Erden A (2020). Evaluation of seventeen patients with COVID-19 pneumonia treated with anakinra according to HScore, SOFA, MuLBSTA and Brescia-COVID respiratory severity scale (BCRSS) scoring systems. J. Med. Virol..

